# Synthesis rates of myosin and actin in skeletal muscle of critically ill patients

**DOI:** 10.1186/2197-425X-3-S1-A179

**Published:** 2015-10-01

**Authors:** I Tjäder, M Klaude, AA Hssain, C Guillet, J Wernerman, O Rooyackers

**Affiliations:** Karolinska University Hospital, Huddinge, Sweden; University Hospital of Clermont-Ferrand, Clermont-Ferrand, France; Clermont University, INRA, Clermont-Ferrand, France; Karolinska Institutet, CLINTEC, Huddinge, Sweden

## Introduction

Critical illness is characterized by a dramatic loss of skeletal muscle mass which comprises to about 10% per week. This loss is mostly driven by an increased muscle protein breakdown, whereas synthesis rates are on average not changed [1]. However, this result limits to total muscle protein and does not elucidate whether different proteins behave differently. We have previously shown that mitochondrial protein synthesis in skeletal muscle of critically ill patients is not different from controls [2].

## Objectives

Here we investigate, in the same patients, whether the major contractile proteins, myosin and actin, behave the same.

## Methods

Seventeen critically ill patients with multiple organ failure and treated in the ICU and ten aged matched metabolically healthy patients undergoing elective surgery were included. Protein synthesis was measured in vivo using a flooding dose of D5-phenylalanine and measurement of the incorporation of this tracer into muscle protein over 90 minutes. Ninety minutes after an iv infusion of the tracer, muscle biopsies were obtained from the vastus lateralis using a Bergström needle. Myosin and actin were isolated from the muscle sample by sequential centrifugations and analyzed for D5-phenyalanine in the proteins by mass spectrometry. Fractional synthesis rates (FSR) were calculated by dividing the protein incorporation into the 2 proteins by the amount of D5-phenyalanine in the precursor pool (plasma). Mann-Whitney U-test was used for statistical comparison. Results are given as median and range.

## Results

All critically ill patients had multiple organ failure with SOFA (sequential organ function assessment) scores between 3 and 12 on the study day. Ages of the groups were comparable (ICU 69; 25-77 years and control 70; 48-76 years). Synthesis rates of myosin were significantly lower in muscle of the critically ill patients (0.79; 0.31-6.95 %/day versus 1.17; 0.95-2.25 %/day; p = 003). No difference in synthesis rates of actin were observed (3.14; 1.58-6.38 %/d versus 2.44; 2.01-4.2; p = 0.05).

## Conclusions

Despite that mixed protein synthesis is on average normal in critically ill patients, this is not the case for the contractile proteins. Synthesis of myosin is decreased and there is a trend for actin synthesis to be upregulated. Possibly this mismatch explains the lower myosin/actin ratios observed in muscle of critically ill patients [3].

## Grant Acknowledgment

Stockholm County Council and Swedish Research Council.
